# Augmented Reality as an Aid to Behavior Therapy for Anxiety Disorders: A Narrative Review

**DOI:** 10.7759/cureus.69454

**Published:** 2024-09-15

**Authors:** Ravi P Rajkumar

**Affiliations:** 1 Psychiatry, Jawaharlal Institute of Postgraduate Medical Education and Research, Puducherry, IND

**Keywords:** augmented reality, panic disorder, generalized anxiety disorder, specific phobia, social anxiety disorder, anxiety disorders

## Abstract

Anxiety disorders are among the most common mental disorders worldwide. These conditions are characterized by excessive anxiety that is difficult to control. In most anxiety disorders, symptoms are triggered by exposure to specific objects or situations. This leads sufferers to avoid such exposures, leading to impaired social and occupational functioning and reduced quality of life. Therapies based on behavioral principles, either alone or in combination with cognitive techniques, are the most effective psychological interventions for anxiety disorders. However, the effectiveness of these therapies may be limited due to a lack of generalization from clinic to real-world settings.

Augmented reality (AR) is a technology that provides an interactive experience by superimposing computer-generated content, often in multiple sensory modalities, on the real world. Emerging evidence suggests that AR may be useful in treating a broad range of mental disorders, including anxiety disorders.

This review examines the evidence for the use of AR-based techniques as an aid to behavioral or cognitive-behavioral therapies for anxiety disorders. The available evidence suggests that this method may offer significant advantages over conventional therapies, particularly in the case of specific phobias, but also in social anxiety disorder. AR can also be combined with other novel technologies to monitor psychophysiological markers of anxiety and its reduction over the course of treatment.

The advantages of AR could be related to its combination of real and simulated content, allowing for better generalization of the benefits of conventional exposure-based therapy. Though the safety, efficacy, and cost-effectiveness of this method need to be confirmed in larger samples, it could lead to a paradigm shift in the way behavioral therapies for anxiety disorders are conceptualized and delivered.

## Introduction and background

Anxiety disorders are the most common mental disorders worldwide, next to depression. According to the most recent Global Burden of Disease study, conducted in 2019, it has been estimated that at least 300 million people, or 3.8% of the world’s population, suffer from anxiety disorders [1]. Based on extrapolations from time trends in the available data, almost 5% of the global population will be diagnosed with an anxiety disorder by the year 2044 [2].

Broadly speaking, anxiety disorders can be classified based on whether they involve a phobic component or not. The term “phobia” refers to an excessive or irrational fear of a particular object, animal, place, or situation, associated with both physical and psychological symptoms of anxiety [3]. Phobias are classified according to the nature of the triggering object or situation. This could be an object, animal, or situation in the external environment (specific phobia), a social situation involving performance or scrutiny (social phobia), or a particular class of situation from which a rapid exit is considered difficult or impossible (agoraphobia). Anxiety disorders without a phobic component include generalized anxiety disorder, in which there is excessive or “free-floating” anxiety or worry related to multiple daily situations, and panic disorder without agoraphobia, in which there are paroxysmal episodes of anxiety without any apparent triggering factor. The distinction between these conditions is not always clear-cut. Many patients may fulfill criteria for more than one anxiety disorder, or have symptoms of both phobic and non-phobic anxiety [4]. Due to these symptoms, persons with anxiety disorders either avoid those situations or objects that “trigger” their anxiety or endure them with difficulty. This leads to significant impairments in social and occupational functioning, and reduced quality of life in several domains; it is estimated that anxiety disorders account for over 28 million disability-adjusted life years (DALY) around the world [5].

Anxiety disorders tend to be chronic and respond only partially to conventional modes of treatment, such as serotonin reuptake inhibitors and psychotherapy [6]. The most widely used psychotherapies are based on behavioral principles derived from conditioning theories, either alone or in combination with cognitive techniques. In the case of phobic anxiety disorders, a central technique is graded in vivo exposure to the anxiety-provoking stimulus or stimuli, accompanied by methods such as relaxation training, cognitive restructuring, and reduction of avoidance [7]. There are several limitations to this approach in clinical practice. First, exposure to an actual feared stimulus may not always be feasible. Second, many patients refuse in vivo exposure due to high initial levels of anxiety, leading to treatment failures. Third, even if exposure therapy is successful in trials or clinic-based settings, it may not generalize to situations or objects encountered in day-to-day life [8]. As a result, exposure-based forms of psychotherapy often have only modest benefits in patients with anxiety disorders, and this is increased only slightly by the addition of cognitive strategies [9,10].

Awareness of the limitations of traditional behavior therapy (BT) and cognitive-behavior therapy (CBT) has led to an interest in the use of novel technologies as aids to therapy in persons with anxiety disorders. Such methods may be particularly useful when conventional therapies are not readily available, or when they are not accepted by patients [11]. One such technique, which has attracted an increasing amount of attention in the past decade, is augmented reality (AR)-based therapy. The term “augmented reality” refers to the combination of real-world and simulated environments through the use of technology-based, multimodal sensory inputs. In contrast to virtual reality (VR), which involves exposure to a simulated world or situation, AR involves a blending of the real and virtual worlds [12]. There are three key differences between AR and VR. First, VR consists entirely of computer-generated, artificial elements, while AR involves a combination of simulated and real elements. In other words, AR lies in the middle of a continuum between the real environment and the simulated environment of VR. Second, VR technology typically restricts the user's movement in space, while AR technology allows the "overlaying" of virtual elements on the real world, allowing for greater mobility and applicability in a wider range of environments. Third, VR hardware and software are designed to maximize the immersive nature of the artificial world, while AR hardware and software are designed to facilitate the superposition of the virtual and the real [13]. Because it allows exposure to novel inputs in a familiar environment, allowing individuals to interact with these inputs in a controlled manner, AR may represent an advance over both conventional and VR-based forms of treatment of mental disorders. A recent review of the literature concluded that AR was potentially useful in a wide range of clinical situations related to mental health. These include training individuals with neurodevelopmental disorders in social and daily living skills, reducing anxiety and distress associated with specific medical procedures, and improving positive mental health through educational interventions [14].

The possibility that AR-based interventions may offer theoretical and practical advantages over existing treatments for anxiety disorder is plausible on several grounds. First, AR-based interventions may allow patients to interact with anxiogenic stimuli that cannot be easily reproduced in a clinic setting. Second, as many AR-based techniques can be controlled through mobile or computer apps, patients may be able to titrate their level of exposure according to their level of comfort and distress, allowing for a smoother form of graded exposure. This could reduce drop-out rates, and even serve as a “bridge” to more challenging forms of exposure in a later, clinic-based setting. Third, as they involve exposure to anxiogenic stimuli in conjunction with the “real world”, they may be superior to clinic-based therapies in terms of generalization. Fourth, AR can concurrently simulate both anxiogenic and supportive stimuli. This may be useful in those patients who engage in “safety behaviors” that interfere with exposure therapy and maintain anxiety symptoms [15,16].

This review will examine the potential for the use of AR in the treatment of anxiety disorders in terms of three key questions. First, is there good-quality evidence that AR-based treatments are effective in managing patients with anxiety disorders? If so, what is their efficacy when compared to conventional treatments? Second, what are the possible physiological and psychological mechanisms associated with symptomatic improvement in patients receiving AR-based therapies for anxiety disorders? Third, are AR-based therapies for anxiety disorders accessible and acceptable to patients and therapists? What are the barriers to their use and acceptance?

## Review

This review was designed and conducted based on standard quality indicators for narrative reviews [17,18]. Relevant, English-language articles on the efficacy, processes of change, and acceptability of AR-based therapies for anxiety disorders were retrieved from the PubMed and Scopus databases. When selecting articles, only original research was included in the review. This research could be in the form of feasibility studies, pilot studies, or controlled clinical trials. Feasibility studies (to assess practical concerns or process variables) were selected if they included either healthy volunteers with subclinical symptoms of anxiety, or patients with anxiety disorder. Pilot and controlled trials (to assess efficacy) were selected only if they included patients with anxiety disorders. This was augmented by a search for conference presentations and other grey literature using Google Scholar [19]. The search terms used were “augmented reality” along with “therapy”, “therapies”, or “psychotherapy”, in conjunction with “anxiety disorder”, “specific phobia”, “social phobia”, “social anxiety disorder”, “panic disorder”, “agoraphobia”, or “generalized anxiety disorder”. Articles published on any date up to August 24, 2024 were included. 

Efficacy of AR-based techniques for anxiety disorders

The earliest report examining the use of AR as an aid to exposure therapy in anxiety disorders was a case report of a young woman with a specific phobia involving cockroaches. She was treated with an intensive, single-session (3 hours), therapist-guided form of exposure therapy. During this session, AR was used to provide exposure by overlaying and moving virtual cockroaches over the patient’s and therapist’s hands. There was also the option of using simulated instruments - a fly swatter and pesticide - to “kill” them. It was found that AR-assisted exposure significantly reduced the patient’s fear and avoidance behavior at the end of the session. These gains were maintained when she was followed up after two months [20]. This technique was subsequently extended to nine patients with cockroach or spider phobia, all of whom reported at least a 40-50% reduction in anxiety after a single session of AR-augmented intensive exposure [21]. In subsequent reports from the same research group, it was found that this technique, when applied to six patients with the same condition, resulted in good outcomes for as long as 12 months [22, 23]. More recently, a combination of AR-based exposure with psychoeducation and cognitive techniques was tested by an independent group of four women with a specific phobia of cats. Three of them reported significant improvement, and gains were maintained for up to three months [24].

Most controlled clinical trials of AR-assisted therapies have involved patients with specific phobias, especially those involving small animals such as insects or moths [25-30]. The results of these studies are summarized in Table 1 below.

**Table 1:** Randomized controlled trials of augmented reality (AR)-assisted behaviour therapy for patients with anxiety disorders Abbreviations: AR, augmented reality; ARET, augmented reality exposure therapy; IVET, in-vivo exposure therapy; WL, waiting list

Study	Diagnosis and sample characteristics	Comparator intervention	Follow-up period	Results	Risk of bias
Wrzesien et al., 2011 [25]	Specific phobia (small animals), n = 12	IVET, single session	Immediate post-session	ARET = IVET on measures of anxiety, catastrophic thoughts, and behavior. IVET > ARET in reducing avoidance.	High risk of bias
Botella et al., 2016 [26]	Specific phobia (spiders), n = 63	IVET, single session	6 months	IVET > ARET on behavioural measures of fear, avoidance; IVET=ARET on self-reported fear immediate post-session. IVET = ARET at 3 and 6 months.	Low risk of bias
Suso-Ribera et al., 2019 [27]	Specific phobia (small animals), n = 91	VRET (n = 28) IVET (n = 31)	Immediate post-session	ARET = IVET = VRET on fear and anxiety. IVET > ARET = VRET on avoidance in patients with higher baseline avoidance.	High risk of bias
Javanbakht et al., 2021 [28]	Specific phobia (spiders), n = 25	WL control (n = 12)	1 month	ARET > WL on fear and behavior post-session, after 1 week and at 1 month.	Some risk of bias
Zimmer et al., 2021 [29]	Specific phobia (spiders), n = 66.	WL control (n = 33)	6 weeks	ARET > WL on fear, disgust, and behavior at 6 weeks	Low risk of bias
Jurcik et al., 2024 [30]	Specific phobia (spiders), n = 55	IVET, single session (n = 18) WL control (n = 17)	1 month	ARET = IVET > WL on spider phobia; ARET = IVET = WL on fear of spiders; ARET > IVET = WL on general anxiety	Some risk of bias

Overall, it was found that AR-assisted exposure therapy (ARET), delivered either as a single intensive session or as a smartphone-based intervention, was comparable to in vivo intensive exposure (IVET) [25, 26] and superior to no treatment at all [28-30]. There was evidence that IVET was slightly better than ARET in reducing avoidance behavior, particularly in those patients where this behavior was prominent at baseline, though a single study found that medium-term (3 to 6 months) outcomes were comparable [26]. Only one report compared ARET with virtual reality-enhanced exposure and found them to be equal in efficacy, but this report was based on secondary analyses of trial data [27]. Trial quality in this field was mixed. Of the six included studies, two were found to be at high risk of bias, two were rated as having some risk of bias, and two were rated as having a low risk.

A single report examined the efficacy of a combination of AR, VR and neurofeedback as an add-on treatment in patients with agoraphobia (n = 3) or social phobia (n = 3). Patients received these sessions once in two weeks over a period of five months. Overall, both groups of patients showed only modest improvement in symptom severity scores (13.4% for agoraphobia and 3.5% for social phobia) [31].

A pilot study examined the efficacy of an AR game, based on the principles of “sandplay therapy” - in which individuals create a “model world” that reflects their emotions and conflicts using toys [32] - in individuals screening positive for generalized anxiety disorder (GAD) [33]. Participants were randomized to the “SandplayAR” game or to a control group. Those who received the AR intervention reported significant improvements on two of 22 items from a self-reported “healing experience questionnaire” when compared to the control group. In view of the lack of a formal GAD diagnosis in participants, the absence of any measure of core anxiety symptoms pre- and post-therapy, and the non-standard therapy model adopted, it is difficult to conclude whether AR-based treatments are effective in GAD based on this study.

Overall, it can be concluded that there is low- to moderate-quality evidence supporting the use of ARET in a subset of patients with specific phobias, namely those with small animal phobias. There is also low-quality evidence that the benefits of ARET in these patients are sustained over a period of 3 to 12 months. “Traditional” IVET may be superior to ARET in some aspects, particularly in patients with more severe phobic avoidance. These results require replication by independent research groups in larger samples [8, 34]. There is insufficient evidence to support the use of AR-based approaches in any other anxiety disorder, though further investigation of its use in agoraphobia and social phobia may be warranted.

Processes associated with symptomatic improvement in AR-based therapies for anxiety disorders

Treatment response during psychotherapy for anxiety disorders is dependent on several factors. Three of these are particularly important. The first is the extent to which exposure in the therapeutic setting is “realistic” - in other words, to what extent it resembles the patient’s real-world fears. The second is the capacity of the exposure paradigm to induce a meaningful level of anxiety, both physical and psychological, in the client. The third, which often determines whether a client cooperates with the prescribed intervention, is their expectations regarding therapy, and to what extent these expectations are satisfied in the therapeutic setting [35].

Measuring these factors is particularly important when designing and evaluating AR-based therapies, which involve a “blending” of real and simulated objects. Such a “blending” may be superior to VR as it is “closer to reality”, but this hypothesis needs to be verified in controlled conditions [36].

Realism

The “realistic” nature of the exposure to a feared object or situation has been assessed both in volunteers and in patients with a clinical anxiety disorder. In a sample of 108 volunteers who reported “discomfort” (not phobia) towards a specific animal, the mean realism score obtained with smartphone- or tablet-based ARET was 2.02 (standard deviation 0.99) out of a maximum of 4, indicating a moderate level of realism and marked variability between subjects. Perceived realism was significantly correlated with distress during an exposure task [37]. Very similar results (mean realism score 2.12-2.26, standard deviation 1.04-1.13) were obtained when comparing smartphone and head-mounted ARET to spiders in 65 healthy young adults [38]. During the development of a mobile application for spider phobia, 7 of 20 healthy volunteers (35%) rated the simulated spiders as being highly realistic [39].

In pilot studies of patients with small animal phobia, participants rated the reality of AR-generated cockroach or spider exposures as being between 8 to 10 of a maximum of 10, or 4 to 7 of a maximum of 7, indicating a fair to high degree of perceived realism. Realism was perceived to increase with the number and movement of simulated animals [21, 40, 41]. In a qualitative report of four patients exposed to a virtual cat during ARET, all participants reported that the simulated animal was “perceived as real”, with only one client commenting on the artificial nature of the cat’s eyes [24]. Realism has been measured in only one controlled clinical trial of ARET. Participants in this trial reported a mean immersion value of 76.9 (standard deviation of 19.1, range 17 to 90) of a maximum of 126 for simulated spiders [29].

Based on this limited evidence, it appears that ARET is perceived as more realistic by patients with a phobic disorder than by healthy controls. In both these groups, there is marked variability in perceived realism, as indicated by the large standard deviations.

Induction of Anxiety

Anxiety has both somatic and psychic components. The somatic or physical component of anxiety can be measured using changes in physiological parameters, such as heart rate and skin conductance, while measurement of the psychic component depends on self-reported, subjective ratings of feelings of anxiety or distress [42]. In the context of AR-based therapy, somatic components of anxiety, such as heart rate (HR), heart rate variability (HRV), or skin conductance (SC), can be measured using appropriate, non-intrusive sensors in combination with specialized software [43].

Psychic Anxiety: In three samples of healthy adult volunteers, exposed to simulated small animals, there was a steady increase in subjective distress, with mean scores rising from 0-1 to 4-5 (out of an average of 10), over the course of graded exposure. This increase was statistically significant, and was more prominent in those who had a prior fear of spiders [37, 38]. In a sample of eight Indonesian patients with cockroach phobia, graded exposure to a single simulated cockroach induced low levels of self-reported anxiety (rated 1-7 on a 10-point Likert-type scale, with a mean score of 3.3). As the number of simulated cockroaches and their movement increased, the level of induced anxiety increased (mean score 5.9), but two of the patients still reported low levels of anxiety [39]. A study comparing AR and VR for the induction of claustrophobia-like anxiety in healthy adults found that both methods induced comparable levels of subjective distress, but scores were low in both cases - 29.2 for AR and 27.6 for VR out of a maximum of 100 units [44].

Somatic Anxiety: In 20 healthy young adults, four of whom reported a fear of spiders, mobile-based AR exposure to spiders or cockroaches resulted in an increase in HR followed by recovery, and an increase in heart rate variation when compared to a neutral stimulus. This effect was more marked in those with a prior history of fear [45]. In a study comparing AR and VR-based induction of anxiety in 34 healthy adults, both methods resulted in a significant increase in SC, no appreciable change in HR, and a trend towards a decrease in HRV. Sub-group analysis revealed that AR produced a greater decrease in HRV than VR [44, 46]. Similar result - increased SC but no significant change in HR - were obtained in two independent samples of healthy adults exposed to AR spiders through a headset or smartphone [37, 38]. Finally, in a controlled clinical trial comparing ARET and in vivo exposure for spider phobia, both treatments led to significant decrease in SC at the 1-month follow-up [30].

In summary, AR for specific phobias appears to be efficacious at inducing psychic anxiety in healthy volunteers as well as in patients, though the effect appears proportional to the baseline level of fear. Only one physiological parameter, skin conductance, appears to increase significantly following AR exposures to phobic stimuli, and appears to normalize after ARET to an extent comparable to that of in vivo exposure.

Expectations

Treatment expectations have been assessed in two controlled clinical trials of ARET, both involving patients with a phobia of cockroaches or spiders. In the first, scores on a measure of expectations regarding efficacy, rationality and satisfactoriness of treatment were comparable in patients receiving in vivo (8.3 ± 1.7) and AR (8.2 ± 1.3) exposure therapy [47]. In the second, a measure of the credibility and positive expectancies related to treatment did not differ significantly between AR and in vivo exposure groups (p = 0.86 for credibility, 0.23 for expectancy) [30]. From this limited evidence, it can be tentatively inferred that expectations regarding a favorable treatment outcome are comparable for ARET and in vivo exposure therapies, but only in patients with specific phobias related to insects.

Acceptability of AR-based therapies for anxiety disorders

Despite their many potential advantages, AR-based treatments for anxiety disorders are a novel form of treatment which may not be acceptable to all clients. Moreover, some adverse effects, such as “cybersickness” - a phenomenon similar to motion sickness that occurs with exposure to AR or VR - are specific to this form of therapy [48]. Cybersickness is characterized by symptoms of nausea, giddiness, fatigue, and headache, and can interfere with a patient's acceptance of AR-based interventions [49]. Both subjective acceptability and adverse effects can interfere with treatment adherence and outcomes, which is why it is important to evaluate the existing evidence pertaining to these variables. It is also important that therapists and other mental health professionals working with anxiety disorders find this mode of treatment acceptable, as they are the ones who would need to recommend and implement it outside research settings.

Acceptability

It has been suggested by some researchers that ARET may be more acceptable than in vivo exposure in conditions such as specific phobias - for example, where the feared animal cannot easily be procured, or where high levels of anxiety interfere with IVET [21]. In a survey of 150 patients with these disorders, 27% said they would refuse IVET, as opposed to only 3% refusing AR- or VR-based exposure [50]. In a pilot study of ARET, where participants felt that AR helped to prepare them for vivo exposure, was safe, and would improve adherence when compared to IVET. These patients also reported certain barriers to the acceptability of ARET, namely the weight of the equipment, a lack of familiarity with the technology, and the limited nature of the AR scenarios [27]. In a randomized controlled trial comparing IVET and ARET, both treatments were equally acceptable. However, those receiving ARET found the treatment less “aversive”, while those receiving IVET considered it to be more “useful” [26].

Two studies have examined the acceptability of ARET by therapists. In a pilot survey of three therapists, only one of whom was familiar with ARET, respondents felt that this method was useful in setting clear goals and completing tasks during a therapy session, was safe to use, and was somewhat useful in enhancing the therapist-client relationship. They also identified certain issues with the usability of the ARET system being tested, which might confuse the patient or lead to a loss of time [25]. A second, larger anonymous survey of mental health experts collected and summarized responses on the perceived strengths and weaknesses of AR-augmented therapies for anxiety or stress-related disorders. The reported strengths of ARET included its adaptability, efficacy, ecological validity, and the ability to precisely control the simulated environment. Reported weaknesses included reduced accessibility, the costs of installation and maintenance, a lack of standardization in existing research, a lack of realism in some cases, and the possibility that technology could create a barrier between patient and therapist [51].

Adverse Events

Reporting of adverse events in clinical trials of ARET has been inconsistent, with most studies not reporting any details on these events [28]. In a pilot study of four patients receiving ARET for specific phobia of cats, two patients reported mild headaches and one reported nausea, similar to the phenomenon of cybersickness described above. These symptoms were not severe enough to cause any drop-outs from the study [24]. In a randomized clinical trial comparing ARET to waiting list controls for spider phobia, the authors stated that no participants in the ARET group reported any adverse events [29]. In another randomized controlled trial comparing ARET and IVET for specific phobia, it was stated that “a few” participants reported tiredness, dizziness, and back pain, but no data was provided on their frequency and severity [26]. There have been no reports of serious adverse events in the available literature on AR-based therapies for anxiety disorders.

Overall, it appears that AR-based therapies are generally well tolerated by patients, and are acceptable to both patients and therapists. However, patients may experience transient adverse effects or difficulties with the equipment, while therapists have flagged concerns regarding usability and cost.

Discussion

The current review was developed to answer three questions regarding the efficacy, mechanisms of change, and acceptability of AR-based therapies for anxiety disorders. Regarding the first of these, which pertains to efficacy, there is evidence from controlled trials that ARET is comparable to traditional exposure therapies in the short- to medium-term management of one specific anxiety disorder, namely specific phobia. In vivo exposure may slightly outperform ARET in those with more severe symptoms. The quality of this evidence can be considered “moderate” using the principles laid down in the GRADE guidelines [52]. There is little evidence on whether AR-based therapies are superior to VR-based ones. Regarding the second, AR-based exposure is perceived as having a moderate degree of realism, with significant variability between subjects, and patients’ expectancies regarding treatment are generally positive. AR-based exposure produces appreciable increases in psychic anxiety and in skin conductance, but not in heart rate. Regarding the third question, ARET is generally acceptable to both patients and therapists, despite the identification of some practical barriers. It has few reported adverse effects, though there has been a tendency to underreport these in the trials published to date.

These findings highlight the strengths and weaknesses of existing research on ARET. Though it is of potential value in a wide range of anxiety and stress-related disorders, it has been systematically evaluated and found effective only for specific phobia [8]. This may reflect the relative ease of developing AR-based exposures to specific stimuli such as insects or other animals. While it is true that developing AR-based simulations for conditions such as social phobia or agoraphobia is more challenging, it is feasible in principle [44,45]. A recent publication has described the development of DJINNI, an exposure-based therapy module for social anxiety disorder that combines AR and VR, and that could be integrated into “conventional” cognitive-behavioural therapy for this disorder [53]. It is likely that, as technology develops and therapist familiarity with AR increases, more such modules will be developed. Such modules would permit not just exposure to feared situations or objects, but training in specific skills to handle anxiety-provoking situations [54]. The testing of these modules in well-designed randomized controlled trials could strengthen the evidence base for the use of ARET in both specific and social phobias. For anxiety disorders not related to a specific, well-defined object - generalized anxiety disorder and panic disorder - the development of AR-based modules may be more challenging. Nevertheless, even in these cases, it may be possible to use AR to produce specific cues that trigger symptoms, or that teach anxiety-reducing skills in a blended environment. Such modules have already been developed and used to allay anxiety in patients outside the psychiatric treatment setting - for example, in patients undergoing surgery, or in students beginning a challenging course [55, 56].

Research into psychotherapy involves the consideration of both “process” and “outcome” factors - in other words, understanding not only whether a therapy works but how it works [57]. In the case of ARET for specific phobias, there is some evidence that this method induces anxiety that is qualitatively similar to that experienced during in vivo treatment. Expectancies regarding the efficacy of ARET are generally positive, and have been found to predict treatment outcomes in one of the published trials [47]. In two trials, measure of the therapeutic alliance were comparable for ARET and IVET [25, 47]. In terms of biological correlates of the therapeutic process, IVET is known to induce changes in several physiological parameters, including heart rate, late positive cortical evoked potentials, and changes in the activity of specific brain regions such as the fusiform gyrus and parahippocampal gyrus [58, 59]. To date, ARET has only been shown to alter skin conductance, but not heart rate. Research on these and related parameters in patients undergoing ARET, particularly in would help in understanding the neurobiological signature “signature” of anxiety induction with this mode of therapy, and whether it is distinct from that seen with IVET. It would also be of interest to know if variables associated with response to IVET, such as low trait anxiety, higher levels of motivation and higher cortisol levels, are predictive of response to ARET - or whether there is a separate profile of patients who would benefit from ARET instead of IVET [60].

Surveys of both patients and therapists have highlighted certain advantages of ARET in the context of specific phobia for small animals. The AR stimuli are perceived as easier to control and better tolerated by clients. However, there is an element of uncertainty among respondents over whether AR or in vivo therapy is more useful [26,50,51]. Though some experts have raised concerns that AR may interfere with the therapist-patient relationship, this has not been borne out by data from controlled trials [44, 61]. More significant concerns relate to the user-friendliness of the available technology, its availability outside research settings, the lack of uniformly validated treatment protocols, the initial costs of the required hardware, the time involved in developing and validating software for the implementation of specific scenarios, and the marketing of AR-based interventions that have not been scientifically tested. Moreover, not enough is known about potential adverse effects, particularly those that are specific to ARET. It is possible that some of these limitations can be overcome through improvements in technology, standardization of AR modules, therapist training, and research on the feasibility of AR-based treatments for anxiety in low- and middle-income countries [51]. These potential advances, along with the research questions flagged above, can aid in the transition from in vivo or VR-based therapies to AR-based ones, and to their expansion to disorders other than specific phobia [62].

Certain limitations of the current review should be acknowledged. The amount of original literature in this field is small. Most published reports arise from a limited number of research centers, meaning that key findings on efficacy and tolerability require replication. These reports have certain methodological limitations, including small sample sizes, potential risks of bias in sample selection and outcome measurement, and significant heterogeneity across studies. The efficacy of AR-based approaches has not been systematically evaluated in any anxiety disorder other than specific phobia, and even in this disorder, it has been tested only in the subset of those with a phobia of small animals. Therefore, these findings cannot be readily extrapolated to patients with other types of anxiety disorder. Few reports include a full enumeration of adverse effects and their frequency. There is also a lack of information on the cost-effectiveness of AR-based therapies. Finally, due to the broad-based nature of this review and the lack of data on several outcomes, a formal meta-analysis could not be carried out.

From a clinical perspective, the main implication of this review is that the use of AR-based exposure can be incorporated into the care of patients with specific phobias, particularly if these involve insects or other small animals. This would be subject to the availability of the requisite technology, as well as to the familiarity of the therapist with the use of AR technology and therapeutic techniques. Based on the available evidence, it is premature to recommend the use of AR-based therapies for other anxiety disorders. From a research perspective, this review highlights the need for further research in those anxiety disorders where an AR-based exposure component would be helpful, such as social anxiety disorder or agoraphobia. The lacunae in the existing literature, mentioned in the previous paragraph, would also need to be addressed by future researchers. Over time, it is hoped that the growth of research in this field would allow more patients to benefit from AR-based alternatives to in vivo exposure, especially in patients where a high level of anxiety of distress precludes traditional exposure therapies. 

## Conclusions

AR-based therapies, particularly ARET, appear to be safe and effective in the management of specific phobias. Despite certain limitations of the available evidence, most controlled trials have found that this form of treatment is roughly comparable to in vivo exposure in terms of efficacy, is accepted by patients, and is generally viewed positively by therapists as well. It may be particularly helpful in those who do not tolerate conventional exposure therapy. The key unanswered questions regarding this treatment modality pertain to its use in other anxiety disorders, its feasibility from an economic or logistical perspective, and the psychophysiological correlates of change during its administration. It is likely that the coming decades will see a significant expansion of both the evidence base for ARET in anxiety disorders, and in its availability in routine clinical care.
